# Do innovative immersive virtual reality simulation videos have a role to play in teaching non-technical skills and increasing preparedness for clinical placements for medical students?

**DOI:** 10.15694/mep.2020.000164.1

**Published:** 2020-08-11

**Authors:** Sushil Pal, Rosalind Benson, Paul Duvall, Vidhi Taylor-Jones

**Affiliations:** 1 School of Medicine

**Keywords:** undergraduate, medicine, education, virtual reality, simulation

## Abstract

This article was migrated. The article was not marked as recommended.

Teaching non-technical skills (NTS) is an important part of the undergraduate medical curriculum. Resource intensive high-fidelity simulation has an established role in this. We developed an innovative series of immersive virtual reality simulation videos for medical students. We found they demonstrated efficacy in teaching NTS and after viewing students felt better prepared for clinical placements.

## Introduction

Teaching non-technical skills (NTS) is an important part of the undergraduate medical curriculum. Resource intensive high-fidelity simulation has an established role in this. We developed an innovative series of immersive virtual reality (VR) simulation videos for medical students. We found they demonstrated efficacy in teaching NTS and after viewing, students felt better prepared for clinical placements.

The GMC published a pivotal report on the UK medical graduates preparedness to practice ( Monrouxe *et al.,* 2014). They highlighted the importance of the development of non-technical skills (NTS) including leadership, situational awareness and the clinical environment, team working and clinical decision-making. They reported variability in the quality of teaching that students experience when traditional learning methods of the apprentice model and junior doctor shadowing were employed. However, the benefit of being an integral team member and the importance of familiarity with the specific working environment was recognised to beneficially facilitate the medical student’s preparedness to practice on graduation.

The community of practice (COP) is a social learning theory embedded within many successful clinical learning environments. Learners, namely medical students initially find themselves on the edge of a COP. As they become more integral to the group and their knowledge increases they move more centrally into the COP. We can enable this transition by equipping students with skills to enhance their legitimacy within the clinical setting. This can be achieved by repeated clinical exposure but situated learning can also be replicated within the simulation setting and in doing so increase a medical student’s legitimacy to practice ( Thomas, Reedy and Gill, 2014).

Using high fidelity simulation to teach NTS has noted success ( Coggins *et al.,* 2017). However, it is expensive, requiring dedicated space and large faculty of trained staff necessitating alternatives to be sought. The educational value of low fidelity simulation has been evaluated in comparison with high-fidelity simulation and has shown consistent improvement in both groups in the teaching of complex clinical and management skills ( Bracq, Michinov and Jannin, 2019).

Increasingly VR technology to teach surgical techniques and clinical anatomy is used. It’s utility in the teaching of NTS has been little explored and as yet there is no comparator study with high-fidelity simulation published ( Norman, Dora and Grierson, 2012).

### Aim

We were interested in whether immersive 360° video simulations of clinical practice can enhance and improve the teaching of NTS and future preparedness for clinical placements following the introduction of VR video simulation to medical students at the University of Liverpool.

## Methods

### The Simulation Programme

In 2016-7 the initial project started as several simulation scenarios recorded using 360° cameras. New scenarios were videoed in the 2017-2018 academic year to improve the authenticity and aesthetics. The scenarios were filmed in a common clinical multi-bedded ward-based environment. Scenario learning objectives were developed based on GMC described domains focussing on NTS such as teamwork, communication and task prioritisation ( Monrouxe *et al.,* 2014).

These scenarios were integral to the development of a simulation programme for the 3 ^rd^ year students, aiming to increase the students’ exposure to simulated practice through the combination of emerging technology and innovative clinical scenarios, otherwise difficult to deliver to large numbers of students. The scenarios provided a unique opportunity to exposure students to situations they rarely face such as blood transfusion errors, and to those scenarios to be experienced in authentic clinical environments with authentic clinical protagonists.

The programme consisted of one high-fidelity simulation session and three lectures based on immersive 360° videos. During the lectures, students would watch the clinical scenarios unfold. Delivered by a highly experienced simulation facilitator and clinician, the scenarios would be interrupted at key points to enable discussion about demonstration of NTS in the style of debriefing a clinical scenario.

On programme completion all students were invited to take part in an anonymous Likert scale questionnaire. Shortly after a focus group was formed which allow thematic analysis to take place from their responses.

## Results/Analysis

101 students responded to the questionnaire, of which 89% had attended all lectures and 82% had attended their high fidelity simulation session.

In all NTS domains assessed, students reported greater understanding of the clinical decision making process (79%), task prioritisation and delegation (69%) and the clinical environment (69%). 71% of students reported feeling better prepared for clinical placement as a result of the programme, stating in part increased confidence in the clinical environment (59%).

Thematic analysis following a focus group interview showed strong concordance of the described themes. Students reported benefiting from the immersive environment of the simulation programme describing the ‘ *safe space*’ for their first exposure to the clinical scenarios as helpful. They found observing the scenarios enabled them to feel they could imitate some of the behaviours displayed by the doctors in the scenes using terms like ‘ *role modelling*’. They found the deconstructive/debriefing elements to the programme meant they developed a more critical approach to their learning following ward placement (see extract 1). These findings are consistent with improving a student’s sense of legitimate peripheral participation.

### Extract 1: Focus group comments

‘I learned that you should be critical as well, in your own practice and in other peoples’ practice.. you have a tendency.. in clinical placement to just watch the ward round and not really take it.. overwhelmed by it.. So, I think that’s helped me see placement in a different way, I don’t just take everything on face value for granted.. I’m actually think, oh, the doctor’s doing this.. maybe he’s stressed, things like that..’

## Discussion

The immersive 360° videos can also be viewed by utilising emerging video hosting software enabling a more individualised experience for students. The school has partnered with a start-up company called Virti™ to host these videos. Through the creation of interactive layers over the videos, student can interact with the content in a unique way on their smartphones, choosing to watch them in both VR and non-VR formats. As the scenario progresses, questions and information boxes appear in the form of annotated layers of text on the screen which the student can choose to interact with as part of their learning experience. Multiple packages can be created from the same scene depending on what the specific learning objectives are.

Next we will embed the scenarios within complete learning packages including links to algorithms and guidelines used in the scenarios. The school also aims to provide VR headsets compatible with most smartphone devices. Further scenarios will be created that look at more diverse clinical environments including general practice, paediatrics and mental health.

## Conclusion

Our research has shown that the development of our unique simulation programme using both VR video simulation alongside traditional high-fidelity simulation has clear educational value in the teaching of NTS, translating into the medical student feeling better prepared for clinical placement.

## Take Home Messages

• Teaching non-technical skills (NTS) is an important part of undergraduate medical curriculum. It is an important part of increasing medical students preparedness for clinical placement.
• High fidelity simulation is resource intensive which consequently limits its accessibility.
• A programme of combined virtual reality video simulation alongside high fidelity simulation offers a novel and effective approach to teaching non-technical skills also enabling the student to access the material outside of the educational setting.

## Notes On Contributors

**Dr Sushil Pal** - Former Clinical Medical Education Fellow at the University of Liverpool and now a Core Anaesthetics Trainee in the Mersey region.

**Dr Rosalind Benson** - Honorary Clinical Medical Education Fellow at the University of Liverpool and a Rheumatology/General Internal Medicine Specialist Trainee in the Mersey region. ORCID: https://orcid.org/0000-0003-2219-6393

**Mr Paul Duvall** - Former Director of Technology Enhanced Learning at the University of Liverpool.

**Dr Vidhi Taylor-Jones** - Director of Simulation at the University of Liverpool and Consultant Anaesthetist at Aintree University NHS Foundation Trust.
