# Do innovative immersive virtual reality simulation videos have a role to play in teaching non-technical skills and increasing preparedness for clinical placements for medical students?

**DOI:** 10.15694/mep.2020.000164.2

**Published:** 2021-09-29

**Authors:** Sushil Pal, Rosalind Benson, Paul Duvall, Vidhi Taylor-Jones

**Affiliations:** 1 School of Medicine; 2 School of Medicine

**Keywords:** undergraduate, medicine, education, virtual reality, simulation, COVID-19, non-technical skills, medical education

## Abstract

This article was migrated. The article was marked as recommended. Background: Teaching non-technical skills (NTS) is an important part of the undergraduate medical curriculum. Resource intensive high-fidelity simulation has an established role in this. Alternative methods of delivering large scale simulation-based education should be considered to help further improve NTS and preparedness for clinical placements of medical students. Emerging technologies such as immersive virtual reality (VR) may have a role in this. Aim: To assess if a VR simulation-based teaching programme enhances understanding of NTS and preparedness for clinical placements in medical students at the University of Liverpool. Methods: A VR simulation-based teaching programme, consisting of 4 sessions of lecture-based simulation and a hi-fidelity simulation session was delivered to 3 ^rd^ year medical students. The lecture-based sessions used pre-recorded, immersive clinical scenarios developed by the School of Medicine, with a focus on NTS. The hi-fidelity simulation session was delivered by local hospital trusts. A survey was sent to all students to assess their understanding of key NTS: decision making, task prioritisation and delegation and how the clinical environment works. Preparedness for clinical placement and confidence in the clinical environment was also assessed. A focus group further explored how students felt towards these NTS, with subsequent thematic analysis.  Results: 101/281 students responded to the survey reporting a greater understanding in all NTS assessed. Students also described feeling better prepared for clinical placements. The focus group reported the programme provided a ‘safe space’ for learning alongside increasing understanding of role modelling and self-awareness.  Discussion: Utilising emerging technology alongside hi-fidelity simulation increased students’ exposure to the clinical environment and enabled exploration of NTS by students. Additional work with larger focus groups will be required to further validate our results. Whilst restrictions are limiting clinical exposure due to the COVID-19 pandemic, we propose that VR simulation-based teaching programmes could provide an alternativeeducational tool.

## Introduction

The General Medical Council (GMC) published a pivotal report on the UK medical graduates’ preparedness to practice ( Monrouxe *et al.,* 2014). They highlighted the importance of the development of non-technical skills (NTS) including leadership, situational awareness within the clinical environment, team working and clinical decision-making. They reported variability in the quality of teaching that students experience when traditional learning methods of the apprentice model and junior doctor shadowing were employed. However, the benefit of being an integral team member and the importance of familiarity with the specific working environment was recognised to beneficially facilitate the medical student’s preparedness to practice on graduation.

The community of practice (COP) is a social learning theory embedded within many successful clinical learning environments ( Wenger and Lave, 1991). Learners, namely medical students initially find themselves on the edge of a COP. As they become more integral to the group and their knowledge increases, they move more centrally into the COP. We can enable this transition by equipping students with skills to enhance their legitimacy within the clinical setting. This can be achieved by repeated clinical exposure but situated learning can also be replicated within the simulation setting and in doing so increase a medical student’s legitimacy to practice ( Thomas, Reedy and Gill, 2014).

Using high fidelity simulation to teach NTS has noted success ( Coggins *et al.,* 2017). The educational value of low fidelity simulation has been evaluated in comparison with high-fidelity simulation and has shown consistent improvement in both groups in the teaching of complex clinical and management skills ( Bracq, Michinov and Jannin, 2019). Increasingly, Virtual reality (VR) technology is used to teach surgical techniques and clinical anatomy. Historically VR’s effectiveness of the teaching of NTS has been little explored but rather the focus has been on ‘useability and acceptability of VR simulation’ ( Bracq, Michinov and Jannin, 2019). Furthermore, in our in our review of the literature we have been unable to find a comparator study with high-fidelity simulation published.

The high cost and resource heavy nature of hi-fidelity simulation provides a compelling argument for educational institutions to find alternative methods of delivering simulation to a large number of students. The combination of VR technology and the principles of simulation may provide such an alternative.

Aim

We were interested in whether immersive 360° video simulations of clinical scenarios can enhance and improve the understanding of NTS and future preparedness for clinical placements, following the introduction of a VR simulation-based teaching programme to medical students at the University of Liverpool.

## Methods

The VR simulation-based teaching programme spearheaded by the Technology Enhanced Learning and Simulation directors was developed during a time of curriculum development in the School of Medicine at the University of Liverpool. A speculative approach by a VR startup company called Virti™ (run by an ex- junior doctor) resulted in the development of software to host this new teaching programme. The content created would initially be used in two ways: as part of standalone interactive learning packages and as part of the simulation programme which, would incorporate existing hi-fidelity simulation run by local hospital trusts. The learning packages would be accessible via a smart phone app and utilise personal VR headsets. This paper will focus on the latter aspect of the project.

The School of Medicine also contracted REAL SPACE LTD™, a film and media company comprising of two University of Liverpool PhD students, that works to support creative freelancers, entrepreneurs and technological start-ups in the VR and augmented reality (AR) space. They filmed each scenario in the simulation suite with their 360° cameras and edited them into final, useable films. Virti™ provided the hosting software and app which was used for the stand-alone learning package. Eight new clinical scenarios were recorded over two days and relied on volunteers from the school’s professional services and teaching faculty to act in the films in various roles i.e. clinical and non-clinical roles where clinical roles were played by clinical staff to help improve authenticity. The editing took approximately 2-4 weeks before final versions were approved by the School of Medicine. The final cost of the project is currently subject to a non-disclosure agreement.

Online polling software, Poll EV™ ( www.pollev.com), was also utilised during the simulation programme as a way for students to engage with content during the sessions. The school had paid for a yearly subscription for use in other areas of the curriculum, thus did not create an additional cost for the project.

### The Simulation Programme

In 2016-17 the initial project started as four simulation scenarios recorded using 360° cameras. New scenarios were filmed in the 2017-2018 academic year to improve the authenticity and aesthetics. The new scenarios were filmed in a common clinical multi-bedded ward-based environment. All of the scenarios were developed by clinicians at the medical school, with core learning objectives forming the foundation for each. Learning objectives were developed based on GMC described domains focusing on NTS such as teamwork, communication and task prioritisation ( Monrouxe *et al.,* 2014).

The programme consisted of one high-fidelity simulation session and four (1 to 2 hour long) highly interactive lecture-based sessions which utilised the immersive 360° videos. The high-fidelity simulation sessions took place within various associated hospital simulation suites. These addressed traditional learning outcomes which focused more on technical and clinical outcomes. During the lectures, students would watch the clinical scenarios unfold and develop. Delivered by a highly experienced simulation facilitator and clinician, the scenarios would be interrupted at key points to enable a discussion and debrief about the NTS demonstrated. Students could discuss the content through open forum or utilise Poll EV to submit answers or opinions which would be displayed to all students, in real time, in the lecture theatre. Table 1 outlines two of the scenarios that were used for these sessions. Both non-technical and clinical learning objectives are also outlined.

**Table 1.** Overview of two scenarios utilised for the lecture-based simulation sessions.

Scenario	Overview	Non-Technical Skill objectives	Clinical objectives
Acute Kidney injury	Foundation doctors are about to start completing tasks following the morning ward round. The nurse informs them of blood results that need to be urgently reviewed and acted upon.	Leadership. Task prioritisation. Communication and handing over.	Management of acute kidney injury. Principles of management of the acutely unwell patient.
Breathlessness	Foundation doctor reviews the investigations of a breathless patient with COPD which shows evidence of metastatic lung cancer. The clinical team needs to inform the patient and relatives of the possible diagnosis.	Breaking bad news. How to approach a ‘Do not attempt resuscitation’ conversation. When to escalate decision making to a senior.	Understand the management of an acute exacerbation of chronic obstructive pulmonary disease. Principles of oxygen therapy.

On programme completion, all students were invited to take part in an anonymous Likert scale questionnaire. The Poll EV online platform was used to host the questionnaire and access link was sent to all students (see Supplementary File 1 for the survey questions). A month later, a focus group took place, where a semi-structured discussion explored students’ understanding of the NTS highlighted in the lecture sessions and the simulation programme as a whole (see Supplementary File 2 for focus group questions). The group consisted of 2 students and was conducted by Dr Vidhi Taylor Jones with input from Dr Sushil Pal, over the course of an hour. The interview was recorded, and the transcripts were used to enable a thematic analysis, using Braun and Clarke’s six phase process ( Braun and Clarke, 2006).

### Target audience

3 ^rd^ year medical students at the University of Liverpool were the targeted audience for the programme. Hospital placements start to form a significant proportion of their curriculum in comparison to earlier years, meaning 3 ^rd^ year students were more likely to have a better baseline understanding of how the clinical environment functions. With no prior exposure to such technology in the curriculum, the lecture-based simulation would complement the hi-fidelity simulation programme already in place for this year group. The scenarios were developed with a view to increase students’ exposure to simulated practice through the combination of emerging technology and innovative clinical scenarios, which would otherwise be difficult to deliver to a large number of students. The scenarios provided a unique opportunity to exposure students to situations they rarely face in undergraduate training and for those scenarios to be experienced in authentic clinical environments with authentic clinical protagonists. Thus, through this, we hoped to legitimise their participation and allow them to become more integral members of a clinical team.

### Data storage and permission

Information sheets were provided to all students and permission was obtained prior to any response, in both the questionnaire and focus group, being used in line with ethical approval guidelines. Data was stored securely on a University encrypted computer and server. No identifiable data was recorded for the questionnaire and names have been omitted during the thematic analysis of the focus group transcripts.

## Results/Analysis

101 students from a cohort of 281 responded to the questionnaire. Of these respondents 90/101 (89%) students had attended all lectures and 83/101 (82%) had attended their high-fidelity simulation session.

In all NTS domains assessed, students reported greater understanding of the clinical decision-making process (80/101, 79%), task prioritisation and delegation (70/101, 69%) and the clinical environment (70/101, 69%). 71% (72/101) of students reported feeling better prepared for clinical placement as a result of the programme, stating in part increased confidence in the clinical environment (60/101, 59%).

Following a focus group interview of two 3rd year students, the authors performed thematic analysis of the transcript. There was clear concordance between the authors in the recognition of a series of themes which we shall further describe.

### A ‘safe space’ for learning

Students reported benefiting from the immersive environment of the simulation programme describing the ‘safe space’ for their first exposure to the clinical scenarios as helpful. One student commented on feeling ‘kind of almost thrown into the clinical environment’ and consequently they stated when asked whether the programme better prepared them for clinical practice ‘to have a safe place for learning I thought was really useful.’ Another commented that having the lecture prior to the high-fidelity simulation session means that ‘everybody gets that exposure to those lectures, where you give us advice on how to deal with those situations before we actually spend the one time that we get to be in that environment’.

The students reported on the first day of a clinical placement it is ‘major sensory overload’ and that ‘these lectures could be really useful at the beginning of each placement’ to provide exposure to the new clinical environment. For some students they received the lecture after the simulation sessions and the focus group felt this was less beneficial.

### Role modelling

Role modelling was a theme noted by the authors. One student noted the programme provided the ‘first exposure of those high-risk situations’ and watching the VR scenarios showed ‘how the leaders approached it’.

They found observing the scenarios enabled them to feel they could imitate some of the behaviours displayed by the doctors in the scenes using terms like ‘role modelling’.

### Self-awareness

The focus group participants described finding the deconstructive/debriefing elements to the programme meant they developed a more critical approach to their learning following ward placement. ‘I learned that you should be critical as well, in your own practice and in other peoples’ practice… you have a tendency…in clinical placement to just watch the ward round and not really take it in…overwhelmed by it…. So, I think that’s helped me see placement in a different way, I don’t just take everything on face value, for granted… I’m actually think, oh, the doctor’s doing this… maybe he’s stressed, things like that…’ These findings are consistent with improving a student’s sense of legitimate peripheral participation but also increasing the student’s self-awareness.

## Discussion

Our experience integrating the novel use of VR technology as part of a simulation programme has been very formative. Recognising the logistical and fiscal challenges that high fidelity simulation delivery to a large cohort of medical students brings, we attempted to look for an innovative adjunct.

Our analysis showed that the majority of students found this programme led to an increased confidence in non-technical skills and the clinical environment and this was echoed in the focus group.

Comments from the focus group suggest that students perceive the clinical environment to be a busy, sometime overwhelming environment. There is a suggestion that exposing students to virtual reality scenarios allows a smoother transition into the clinical environment ( Cleland *et al.,* 2016). It gives the students an idea of what to expect; the sights, sounds, languages and behaviours which student might encounter are all novel experiences for earlier years medical students.

**Figure 1.**
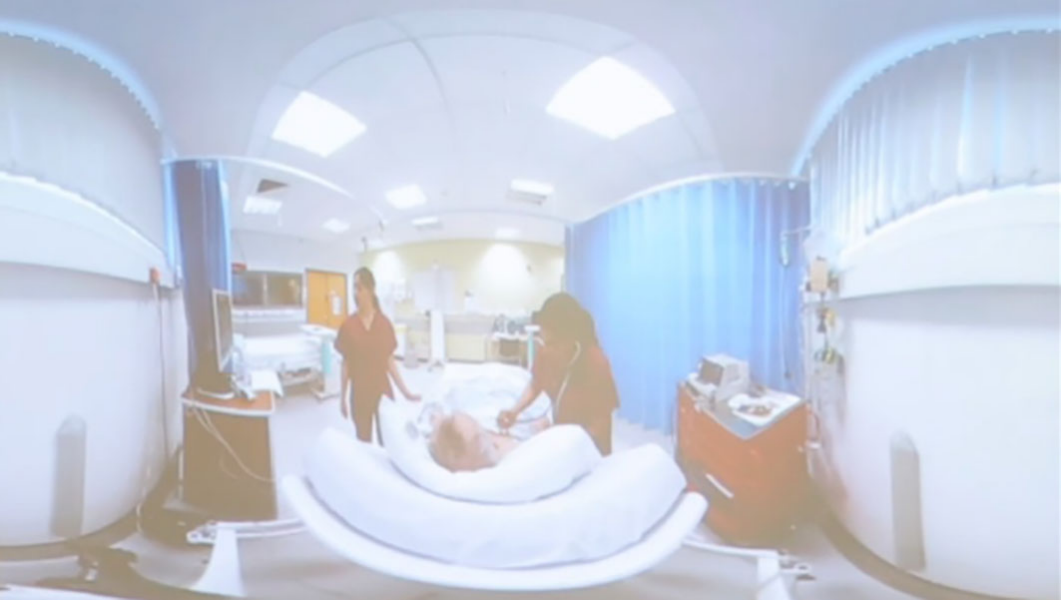
An example of the fisheye view that students would see during the lecture-based sessions.

However, there were limitations both to the programme but also to our evaluation. As with any teaching programme the proficiency of the teacher impacts upon the potential learning of the student ( Sutkin *et al., *2008). Our sessions were delivered by highly experienced lecturers who were not only comfortable delivering the lecture content but doing so whilst managing the technical requirements of the session which included intermittently pausing the video, using interactive polling software and providing different views of the scene (an option provided by filming with the fisheye lens, see Figure 1 for an example of this view).

The optics experienced by the students whilst watching the scenarios unfold were very good. However, displaying the videos on a large screen does not enable the student to fully immerse themselves in the VR technology to the extent that viewing through their own headset would. The Virti™ platform is designed for both individual and collaborative viewing, both of which provide differing learning experiences. Our programme harnessing the collaborative viewing of scenarios enabled large group facilitated discussion on non-technical skills.

We received a questionnaire response rate of 36%, this was despite advertising it prior to the lecture and a reminder at the end of the lecture. Again, the number of students who volunteered to participate in our focus groups was very small. Reflecting on the reasons for this, the focus groups took place when most of the year group was revising for upcoming exams and this likely had a large impact on those volunteering. In the future, we shall time our evaluation more carefully alongside the students’ timetable, but close enough to the programme to maintain validity. Recognising these limitations, the findings of the questionnaire were echoed in detail in the focus groups and all authors found concordance between the themes they identified. To draw more concrete conclusions from this piece of exploratory work, evaluation of the programme with further focus groups would be useful.

We were unable to perform more complex statistical analysis of our questionnaire results because of the way that the data from Poll EV™ was extracted. In further analysis, using different polling software would be preferable.

The project looked at the use of the VR scenarios as an adjunct to high fidelity simulation. The impact of this method of delivering simulation-based education on students learning needs more research, however, we can certainly say that this method provides a more cost-effective way of exposing students to scenario-based learning, which demonstrates, in real time, the synthesis between clinical, communication and non-technical skills. In the context of the COVID-19 pandemic we think there is increased need for innovative approaches to simulation programmes. The practicalities of running simulation sessions are much more complex and we would propose that these VR simulation scenarios provide valuable exposure to a simulated ward environment when, due to COVID-19 related restrictions, clinical placements are more fraught with organisational complexity. There have been a number of recent papers describing online simulation as an adjunct or replacement to face-to-face simulation which could be deemed too high risk for leaners and educators ( Castro and Lucchetti, 2020; Patel *et al.,* 2020).

Next, we will embed the scenarios within complete learning packages including links to algorithms and guidelines relevant to the scenarios. The school also aims to provide VR headsets compatible with most smartphone devices. Further scenarios will be created that look at more diverse clinical environments including general practice, paediatrics and mental health.

## Conclusion

Our research has shown that the development of our unique simulation programme using VR video simulation alongside traditional high-fidelity simulation has clear educational value in the understanding of NTS, translating into the medical student feeling better prepared for clinical placement.

## Take Home Messages

• Teaching non-technical skills (NTS) is an important part of undergraduate medical curriculum. It is an important part of increasing medical students’ preparedness for clinical placement.
• High fidelity simulation is resource intensive which consequently limits its accessibility.
• A programme of combined virtual reality video simulation alongside high fidelity simulation offers a novel and effective approach to teaching non-technical skills also enabling the student to access the material outside of the educational setting.
• With current limitations on clinical placements for medical students due to the COVID-19 pandemic, we propose that VR simulation scenarios could have an increasing role in medical education.

## Notes On Contributors

**Dr Sushil Pal** - Former Clinical Medical Education Fellow at the University of Liverpool and now a Core Anaesthetics Trainee in the Mersey region.

**Dr Rosalind Benson** - Former Clinical Medical Education Fellow at the University of Liverpool and a Rheumatology/General Internal Medicine Specialist Trainee in the Mersey region. ORCID: https://orcid.org/0000-0003-2219-6393

**Mr Paul Duvall** - Former Director of Technology Enhanced Learning at the University of Liverpool.

**Dr Vidhi Taylor-Jones** - Director of Simulation at the University of Liverpool and Consultant Anaesthetist at Liverpool University Foundation Trust.

## Supplementary Files

Supplementary file 1 - Sim med ed publish Version 2.docx
