# Supplementary material for: Do innovative immersive virtual reality simulation videos have a role to play in teaching non-technical skills and increasing preparedness for clinical placements for medical students?
Source: MedEdPublish (2016). 2021 Sep 29;9:164. Originally published 2020 Aug 11. [Version 2] doi: 10.15694/mep.2020.000164.2 (PMC10423946; doi:10.15694/mep.2020.000164.2)
Supplement: Peer review history

# Peer review: Reviewer response for version 2

**DOI:** 10.21956/mep.20275.r31475

**Published:** 2021-10-08

**Authors:** Richard Hays

**Affiliations:** 1 James Cook University

This review has been migrated. The reviewer awarded 4 stars out of 5 The authors have addressed the comments of reviewers of the original version. The piloting and evaluation of VR in medical education is most welcome, particularly in the broader range of skills included in this study. The medium appears to have great potential, particularly in the post-COVID-19 world, and its use is likely to be extended widely. I think that the evaluation is rather weak, but that may not matter at this stage. I am sure that the technology would be exciting, but am not sure it can replace/prepare/augment interaction with SPs and real patients. It will be interesting to see more reports like this.

Reviewer Expertise:

NA

No decision status is available

# Peer review: Reviewer response for version 2

**DOI:** 10.21956/mep.20275.r31474

**Published:** 2021-10-02

**Authors:** Ken Masters

**Affiliations:** 1 Sultan Qaboos University

This review has been migrated. The reviewer awarded 4 stars out of 5 I am pleased to see that Version 1 of the paper has been substantially revised to form an excellent paper that gives the details that readers require. Although there are some minor issues (e.g. I think Figure 1 would be better placed in the Results, rather than the Discussion, and I would like to have seen a stronger Conclusion), the paper is a good read and a useful contribution to the field.One suggestion for future focus groups: the opening questions should not be so pointed (Yes/No) (even though they have an exploratory question that follows).Overall, though, a good read.

Reviewer Expertise:

NA

No decision status is available

# Peer review: Reviewer response for version 1

**DOI:** 10.21956/mep.19138.r27656

**Published:** 2020-11-07

**Authors:** Ken Masters

**Affiliations:** 1 Sultan Qaboos University

This review has been migrated. The reviewer awarded 2 stars out of 5 The paper deals with immersive virtual reality simulation videos in teaching non-technical skills and increasing preparedness for clinical placements to medical students. While the research appears to have been useful, the paper does need a great deal of work. So much so that it is difficult to assess the value of the research itself.I would recommend a Version 2 of the paper, in which the authors address these issues:• The Abstract is far too short and uninformative. It should be properly structured in the same format of the paper, and then give the most important results and findings.• The first paragraph of the Introduction is a repeat of the Abstract, and should be removed.• “It’s utility in the teaching of NTS has been little explored and as yet there is no comparator study with high-fidelity simulation published (Norman, Dora and Grierson, 2012).” This statement should be re-phrased, as it implies that the Norman et al. reference supports the statement that “and as yet there is no comparator study with high-fidelity simulation published”. If this was the case in 2012, it might not necessarily true 8 years later. In addition, it would be safer if the authors wrote that they could not find such a study (as such a study may have been published, but the authors missed it in their search).• There is an error in the Norman et al. reference (The words “Simulation fidelity” have erroneously been added to the title).• In the Methods, far more information is required about:o The technical information about the construction of the videos (including partnering with Virti, costs (in both time and money)) should be given in the Methods (not the Discussion). In fact, most of the Discussion’s first paragraph should go into the Methods.o Given that Virti appears to be a private company, their involvement needs to be explicitly described (what does “partnered” in this study refer to?)o The students, their stage of study (not just “3rd-year”), previous exposure to similar technologies, etc.o The survey: how many questions, how delivered (if online, details, not just “online”). It would be best to supply a copy of the questionnaire.o Focus group: How conducted, by whom, how many participants, how long. It would be best to supply a copy of the initial questions.o Theming: process of theming, including software (if any) used.o Data storage and anonymisation.• In the Results, again, far more information is required:o Student demographics of both the survey and the focus group.o Raw numbers and percentages (even though the total was 101, it’s best to stay with the convention).o Any correlations between the variables, especially demographics and answers to questions, groups (e.g. lectures and session) and the answers to the questions, and statistically significant differences.o Focus group: The data from the focus group should be given properly in themes, with each theme supported by 1-2 quotations.• The Discussion, unfortunately, is lacking. Once the first paragraph has been moved to the Methods (where it should be), the Discussion consists only of looking forward. There is no discussion of the results, relating them back to the literature, no Limitations, etc.• There are quite a few language and punctuation errors in the paper. Sometimes, they are minor irritations, but sometimes, they interfere with the meaning. I would strongly recommend that the authors perform another careful proof-read of the paper.So, while the research appears interesting, I feel that the authors have missed a golden opportunity to write it up as a strong and coherent research paper. I look forward to reading Version 2 in which these issues are addressed.

Reviewer Expertise:

NA

No decision status is available

# Peer review: Reviewer response for version 1

**DOI:** 10.21956/mep.19138.r27654

**Published:** 2020-08-26

**Authors:** P Ravi Shankar

**Affiliations:** 1 American International Medical University

This review has been migrated. The reviewer awarded 3 stars out of 5 This is an interesting article about implementing a mixed educational program utilizing a high-fidelity simulation and virtual reality sessions. Virtual reality can play an increasingly important role in medical education. The cost of VR hardware is decreasing and as the authors mention VR can be accessed through smartphones and VR glasses. One of the issues to be addressed in a revised version is to provide a greater description of what was done during the intervention, what was measured, how it was measured and what was found. Readers can benefit from greater details about what was addressed during the high-fidelity simulation and the topics addressed during the virtual reality (VR) sessions. How were the VR sessions conducted in the classroom? More details about the questionnaire and how the focus groups were conducted and how the data was analyzed will be helpful. How many focus groups were conducted? What were the themes which emerged? Greater details about the company called ‘Virti’ and which topics will be addressed and more information about the videos will be of interest.

Reviewer Expertise:

NA

No decision status is available

# Peer review: Reviewer response for version 1

**DOI:** 10.21956/mep.19138.r27655

**Published:** 2020-08-25

**Authors:** Carol Quinn

**Affiliations:** 1 University of Glasgow

This review has been migrated. The reviewer awarded 4 stars out of 5 important contribution in recognising medical students are on the edge of a community of practice and utilisation of technology to enhance learning. Clear outcomes with appropriate measurement. Practical implications identified.

Reviewer Expertise:

NA

No decision status is available
